# Clinical Utility of Comprehensive Genomic Profiling for Advanced Non‐Small Cell Lung Cancer: A Single‐Center Retrospective Study

**DOI:** 10.1111/1759-7714.70304

**Published:** 2026-06-02

**Authors:** Shuji Murakami, Yukihiko Hiroshima, Seigo Katakura, Tetsuro Kondo, Terufumi Kato, Tomoyuki Yokose, Haruhiro Saito

**Affiliations:** ^1^ Department of Thoracic Oncology Kanagawa Cancer Center Yokohama Japan; ^2^ Department of Cancer Genome Medicine Kanagawa Cancer Center Yokohama Japan; ^3^ Department of Pathology Kanagawa Cancer Center Yokohama Japan

**Keywords:** biopsy, ErbB receptors, high‐throughput nucleotide sequencing, mutation, non‐small cell lung carcinoma

## Abstract

**Background:**

For advanced non‐small cell lung cancer (NSCLC), biomarker testing using multiplex companion diagnostics has become the standard of care. However, challenges remain in detecting rare mutations and resistance mechanisms after targeted therapy. We evaluated the prevalence of druggable genetic aberrations and the clinical utility of comprehensive genomic profiling (CGP) performed after completion of standard treatment in real‐world practice.

**Methods:**

This retrospective study analyzed 108 patients with advanced or recurrent non‐small cell lung cancer who underwent comprehensive genomic profiling testing between January 2019 and December 2023 at the Kanagawa Cancer Center Hospital, Japan. Profiling was conducted using the OncoGuide NCC Oncopanel System, FoundationOne, and FoundationOne Liquid companion diagnostics.

**Results:**

Druggable genetic aberrations were detected in 37% of the patients, with epidermal growth factor receptor mutations being the most common. Resistance mechanisms were identified in 9 of 19 patients who underwent re‐biopsy after targeted therapy. Non‐smokers had a significantly higher rate of druggable mutations (55.6%) than did smokers (27.8%). Treatment based on comprehensive genomic profiling findings was recommended for 35.2% of the patients; however, only 16.6% received the suggested therapy. In a landmark survival analysis, patients who received genomically matched therapy demonstrated a trend toward prolonged overall survival compared with those who did not (log‐rank *p* = 0.054).

**Conclusions:**

CGP testing can identify actionable mutations missed by conventional diagnostics and may provide additional therapeutic opportunities in NSCLC.

## Graphical abstract

Comprehensive genomic profiling (CGP) identified druggable mutations in 37% of patients with advanced non‐small cell lung cancer including resistance mutations after targeted therapy. Non‐smokers showed a significantly higher detection rate. CGP testing provided clinically meaningful treatment opportunities beyond conventional biomarker tests, supporting its utility in routine practice.

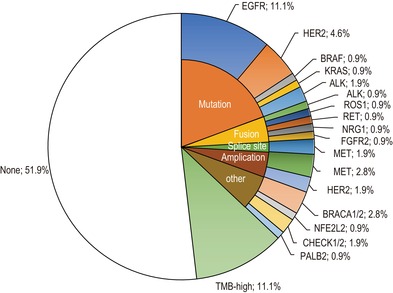

**Abbreviations**
ALK: anaplastic lymphoma kinase
CGP: comprehensive genomic profile
CI: confidence intervals
EGFR: epidermal growth factor receptor
EP: expert panel
MET: mesenchymal epithelial transition
NGS: next‐generation sequencing
NSCLC: non‐small cell lung cancer
NTRK: neurotrophic tyrosine receptor kinase
ODxTT: Oncomine Dx Target Test
PR: partial response
SD: stable disease

## 1 Introduction

In clinical practice, the identification of genomic alterations is essential before making treatment decisions for patients with advanced non‐small cell lung cancer (NSCLC). The development of novel targeted therapies for actionable genomic alterations has opened a new era in the management of advanced NSCLC and has improved survival outcomes [1]. Missing driver mutations can cause patients to miss the opportunity to receive treatment to prolong their survival [2, 3]. Therefore, it is always a challenge to identify driver mutations with as few omissions as possible.

The main nine driver mutations targeted in advanced NSCLC are the following: epidermal growth factor receptor (*EGFR*)‐mutations, anaplastic lymphoma kinase (*ALK*)‐fusions, ROS1 proto‐oncogene (*ROS1*)‐fusions, *KRAS* mutations, *BRAF* V600E mutation, mesenchymal epithelial transition (*MET*) exon 14 skipping, *RET* fusions, human epidermal growth factor receptor 2 (*HER2*) mutations, and neurotrophic tyrosine receptor kinase (*NTRK*) fusions. Moreover, the therapeutic effects of molecular‐targeted drugs against driver gene mutations are not permanent, and the problem of resistance cannot be avoided. Consequently, the search for resistance gene mutations after each molecular‐targeted drug treatment and the development of treatments for resistance mutations are progressing. Information on drug resistance may help guide treatment choices and provide options for clinical trials.

Single‐plex tests, such as real‐time polymerase chain reaction (PCR) and fluorescence in situ hybridization (FISH), have been used to detect single‐gene mutations; however, they have recently been replaced by multiplex tests using next‐generation sequencing (NGS) and real‐time PCR, which can detect multiple genomic alterations in one examination [4]. The Oncomine Dx Target Test (ODxTT; Thermo Fisher Scientific, Waltham, MA), which simultaneously detects 46 cancer‐related genes, was the first NGS panel for NSCLC testing and was granted reimbursement coverage in Japan in June 2019 [4]. The AmoyDx Pan Lung Cancer PCR Panel (AmoyDx PLC panel; Amoy Diagnostics Co. Ltd., Xiamen, China), which simultaneously detects nine NSCLC‐related genes, is a multiplex PCR panel granted reimbursement coverage in Japan in January 2022 [5]. Multiplex tests are rapidly gaining popularity; however, certain concerns have emerged. Discordance between the tested panels due to detectable variants has been reported [5, 6]. This is a limitation of hotspot panels that can only detect mutations in targeted hotspots [4].

Since 2019, several hybrid, capture‐based NGS tests, including the FoundationOne CDx (F1CDx), NCC OncoPanel, and F1LCDx, have been approved as Comprehensive Genomic Profile (CGP) tests in Japan [4]. These CGP tests can detect mutations, amplifications, homozygous deletions, and rearrangements in target cancer genes across the full coding region of the target gene in each panel. Consequently, the CGP testing using the hybrid capture method is expected to identify rare genetic abnormalities that cannot be detected using hotspot panel testing. Additionally, elucidation of the resistance mechanisms of molecular targeted drugs is anticipated. We, therefore, aimed to evaluate the prevalence of druggable genetic aberrations and the usefulness of a CGP panel conducted after the completion of standard treatment for NSCLC in real‐life clinical practice.

## 2 Materials and Methods

### 2.1 Patients and Clinical Data

We retrospectively reviewed the medical records of patients with advanced or recurrent NSCLC who underwent CGP testing between January 2019 and December 2023 at the Kanagawa Cancer Center Hospital, Japan. The following clinical parameters were obtained: age, sex, smoking history, number of prior lines of therapy, previous clinical biomarker tests such as the Oncomine Dx Target Test Multi‐CDx, AmoyDx Lung Cancer Multi‐Gene PCR Panel, single‐plex tests, previously detected driver mutations, and previous treatment with molecular‐targeted therapy. In addition, data were collected on whether the patients received the recommended therapy after the expert panel and whether they benefited from the therapy.

### 2.2 CGP Test and Expert Panel (EP)

The CGP tests used in this study were the OncoGuide NCC Oncopanel System (Sysmex Corporation, Hyogo, Japan), FoundationOne CDx (F1CDx; Foundation Medicine, Cambridge, MA), and FoundationOne Liquid CDx (F1LCDx; Foundation Medicine, Cambridge, MA), all of which are approved by the Japanese National Health Insurance. The type and timing of the CGP tests were determined by the attending physician based on clinical judgment and patient preferences. The tissue specimens were selected based on the attending physician's request and the pathologist's assessment. All CGP tests of tissue specimens were performed on formalin‐fixed paraffin‐embedded samples. In cases with no pre‐existing driver gene mutations, specimens from the initial diagnosis were subjected to CGP testing. If there was insufficient tissue, a re‐biopsy specimen was used. For cases in which molecular‐targeted drugs were used for driver gene mutations, re‐biopsy specimens were primarily submitted for CGP testing after the use of these drugs. CGP testing using liquid biopsy was performed at the discretion of the attending physician when the specimen volume was deemed insufficient for CGP testing or when specimen collection was difficult.

The CGP test results for all cases were reviewed by the EP of the Molecular Tumor Board, which consisted of experts from various fields including oncologists, geneticists, pathologists, bioinformaticians, and genetic counselors. The evidence levels for therapeutic efficacy were categorized from A to F following the guidelines of the Japanese Society of Medical Oncology, Japan Society of Clinical Oncology, and Japan Cancer Association [7]. The turnaround time was defined as the time required to submit the inspection to the EP.

### 2.3 Actionable and Druggable Genomic Aberration and Tumor Mutation Burden (TMB)‐High

Genetic abnormalities were defined as druggable if they had an evidence level of C or higher according to the Cancer Cell Annotation Tool (C‐CAT) evidence‐level classification or if they were targeted in Phase II or III clinical trials. Genetic abnormalities with an evidence level of D or higher or those targeted in Phase I or higher clinical trials were defined as actionable genetic mutations. TMB‐high refers to tumors with a high number of mutations per megabase of DNA, whereas in FoundationOne testing, TMB‐high is defined as having 10 or more mutations per megabase.

### 2.4 Statistical Analysis

The association between the percentage of druggable genetic aberrations detected and each patient's background was examined using the Fisher's exact test. Druggable aberration rates were summarized using frequencies and 95% confidence intervals (CIs). Overall survival (OS) was evaluated using a landmark approach. The date of the EP review was selected as the landmark time point because therapeutic recommendations based on CGP test results were formally determined at that time. Patients who died before the EP review (*n* = 4) were excluded from this survival analysis. Post‐EP OS was defined as the time from the EP review to death from any cause or the last follow‐up. Patients who were alive at the last follow‐up were censored. Survival curves were estimated using the Kaplan–Meier method and compared using the log‐rank test. Statistical significance was defined as *p* < 0.05. Statistical analyses were performed using EZR (Easy R) analysis software version 1.61 (Department of Hematology, Saitama Medical Center, Jichi Medical University, Saitama, Japan) [8].

## 3 Results

### 3.1 Patient Characteristics and Driver Gene Mutation Background

**FIGURE 1:**
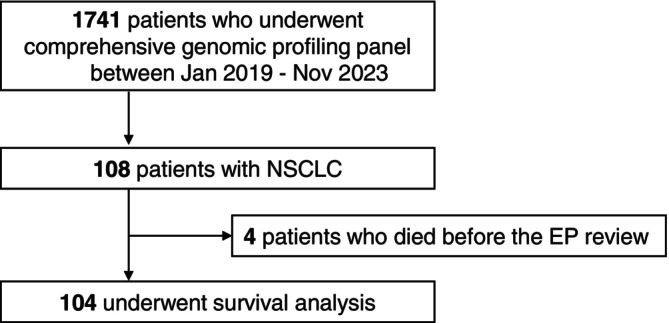
Consort diagram.

A total of 108 patients with advanced or recurrent NSCLC who underwent CGP testing were identified (Figure 1). Table 1 shows the baseline characteristics of the patients. The median age of the patients was 66 (range: 29–90) years; 63 (58.3%) were males, 36 (33.3%) were never smokers, 90 (83.3%) had adenocarcinoma, and the median number of pretreatment lines was three (range: 0–9). A total of 98 and 29 patients underwent genetic biomarker testing and multiplex testing, respectively. Tissue samples were submitted in 86 cases including 47 archival samples from initial diagnoses and 39 samples from re‐biopsies. The median sample storage period was 7 (range: 0.2–134.1) months. Among the genomic profiling tests, F1CDx (*n* = 82; 75.9%) was the most frequently performed, followed by F1LCDx (*n* = 22; 20.4%). Twenty‐seven patients were identified as having driver gene mutations using approved companion diagnostics. Nineteen patients underwent either a re‐biopsy or liquid biopsy after the use of molecularly targeted drugs for driver gene mutations. Fourteen of these re‐biopsies or liquid biopsies were performed after the use of EGFR‐TKIs, and two were performed after the use of ALK inhibitors.

**TABLE 1:** Patient characteristics.

	All patients (*N* = 108)	%	After TKI (*N* = 19)	%
Age (median, range)	66 (29–93)		66 (41–77)	
Sex				
Male	63	58.3	10	52.6
Female	45	41.7	9	47.4
Histology				
Adenocarcinoma	90	83.3	19	100.0
Squamous cell carcinoma	9	8.3	—	—
Other	9	8.3	—	—
Smoking history				
Never	36	33.3	11	57.9
Ever	72	66.7	8	42.1
Sampling method				
Surgery	39	36.1	2	10.5
Biopsy	47	43.5	8	42.1
Liquid	22	20.4	9	47.4
Number of pre‐treatment lines	3 (0–9)		3 (1–9)	
Biomarker tests performed				
Multiplex	29	26.9	5	26.3
Single‐plex	69	63.9	14	73.7
None	10	9.3	—	—
Type of CGP				
FoundationOne CDx	82	75.9	10	52.6
OncoGuide NCC OncoPanel	4	3.7	—	—
FoundationOne Liquid CDx	22	20.4	9	47.4
Sample used for CGP				
Archival tissue	47	43.5	—	
Re‐biopsy	39	36.1	10	52.6
Sample storage period (months)	7.7 (0.2–134.1)		0.9 (0.3–12)	
Turnaround time (days)	29 (21–49)			
Pre‐defined driver mutations	27	25.0		
EGFR	17	15.7	14	73.7
ALK	2	1.9	2	10.5
ROS1	3	2.8	1	5.3
MET	4	3.7	2	10.5
HER2	1	0.9	0	0

Abbreviations: ALK, anaplastic lymphoma kinase; CDx, companion diagnostic; CGP, comprehensive genomic profiling; EGFR, epidermal growth factor receptor; MET, mesenchymal–epithelial transition factor; NGS, next‐generation sequencing; ROS1, c‐Ros oncogene 1; TKI, tyrosine kinase inhibitor.

**FIGURE 2:**
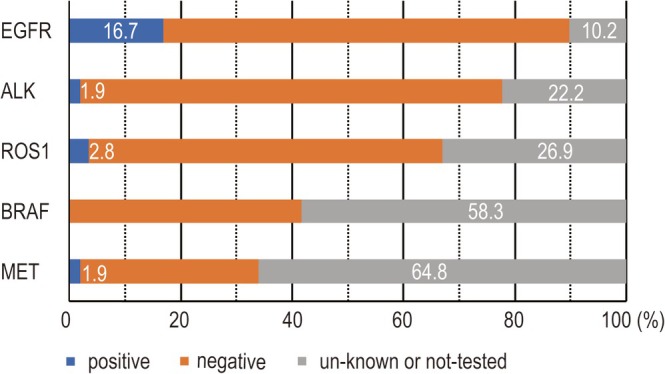
Clinical biomarker test results prior to CGP.

Figure 2 shows the results of the clinical biomarker tests performed prior to CGP testing. In all groups of patients, *EGFR mutations were tested in 89.8% of patients, with 16.7% testing positive; ALK fusions were tested in 77.8%, with 1.9% testing positive; and ROS1 fusions were tested in 73.1%, with 2.8% testing positive*. In contrast, BRAF and MET skipping mutations were detected in less than 50% of cases, with only 41.7% for BRAF and 35.2% for MET.

### 3.2 Druggable Genomic Aberration and TMB‐High

**FIGURE 3:**
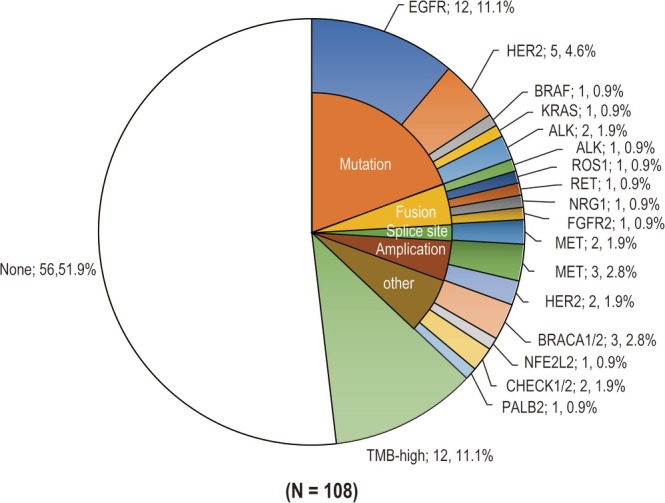
Druggable genomic aberrations detected by CGP.

Figure 3 illustrates the distribution of druggable genomic aberrations among patients. Overall, 37% of patients (*n* = 40) had druggable mutations including various types of genomic aberrations such as mutations, fusions, splice site mutations, and amplifications. The most common druggable genomic mutation identified was EGFR, which was found in 12 patients (11%). This was followed by HER2 mutations in five patients (5%) and BRAF and KRAS mutations in one patient. The ALK mutations observed in two patients (2%) were both G1202R mutations, which are thought to be resistant to ALK inhibitors. ALK, ROS1, RET, NRG, and FGFR2 fusions were found in one patient, whereas MET skipping mutations were found in two patients (2%). Additionally, MET amplification was seen in three patients (3%) and HER2 amplification in two patients (2%). Genomic mutations in BRCA1/2, NFE2L2, CHEK1/2, and PALB2, which are biomarkers for PARP inhibitors covered for off‐label use, were found in seven patients (7%). Six of the seven patients did not exhibit a clear clinical or family history of diseases associated with these variants. Genetic counseling was conducted for three of the 108 patients (one with CHEK2, one with ATM, and one with RAD51) with pathogenic or presumed germline pathogenic variants. TMB‐high was observed in 11% of patients.

### 3.3 Newly Detected Driver Mutation and Resistance Mutations

**FIGURE 4:**
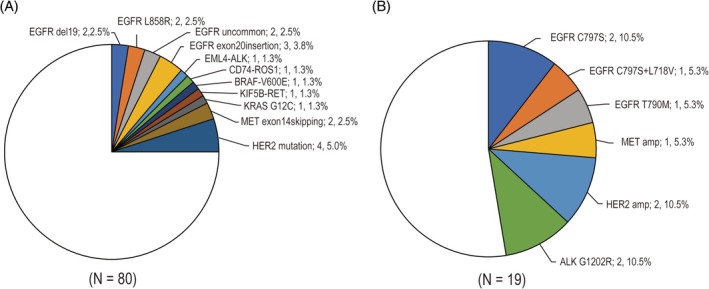
(A) Driver oncogenes newly detected via comprehensive genomic profiling assays. (B) Resistance mutations detected by CGP after molecular targeted therapy.

Of the 80 patients with previously undetected driver gene mutations, the CGP testing newly detected driver gene mutations in 25 patients (Figure 4A). Among patients who were initially negative for EGFR mutations by conventional biomarker testing (*n* = 78), the CGP testing newly identified EGFR mutations in eight patients (10.3%). The analysis revealed that EGFR mutations were prominent, with EGFR del19 and EGFR L858R detected in 2.5% of the patients. Additionally, uncommon EGFR mutations were found in 2.5% of the cases, whereas EGFR exon20 insertions were identified in 3.8%. Additionally, five patients exhibited HER2 mutations, two displayed MET exon 14 skipping mutations, and one each presented with EML4‐ALK, CD74‐ROS1, BRAF V600E, KIF5B‐RET, and KRAS G12C mutations.

Of the 19 patients who underwent re‐biopsy after molecular targeted therapy for driver mutations, nine patients had some genomic aberrations associated with resistance (Figure 4B). Among the 14 patients previously treated with EGFR‐TKIs, acquired resistance‐associated mutations were identified in four patients (28.6%). The prevalence of C797S mutation in patients who acquired resistance to EGFR‐TKIs was 22% (3/14). The EGFR T790M mutation was confirmed in one patient after first‐generation EGFR‐TKI treatment. Other resistance mechanisms were identified in one patient with MET amplification and in two patients with HER2 amplification.

**FIGURE 5:**
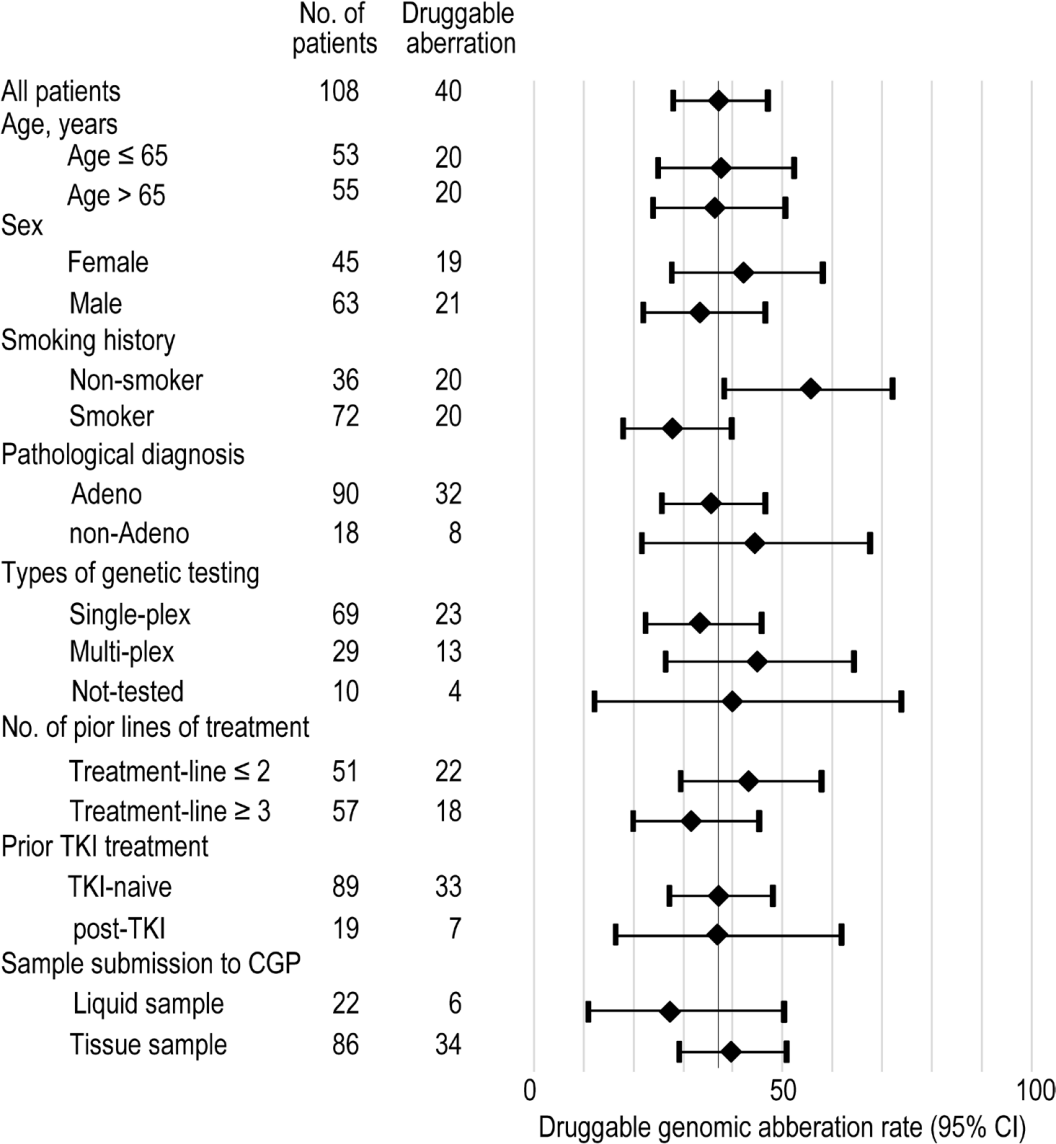
Subgroup analysis of druggable genomic aberration rate.

Druggable aberration rates were similar across the subgroups based on all baseline patient characteristics, except for smoking history (Figure 5). The druggable aberration rate was 55.6% (95% CI, 38.1%–72.1%) in patients with no smoking history, significantly higher than 27.8% (95% CI, 17.9%–39.6%) in patients with a smoking history (*p* = 0.0063).

### 3.4 Drug Accessibility Rate and Clinical Response

**FIGURE 6:**
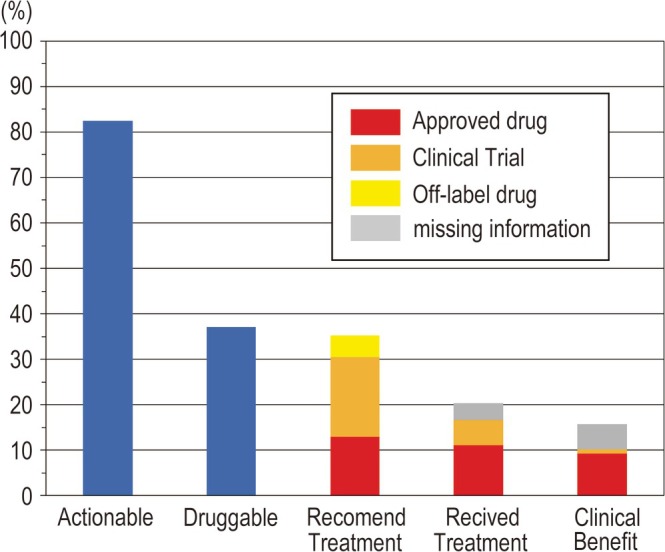
Detected druggable genomic aberration rate, expert panel recommended treatment rate, and drug accessibility rate.

**FIGURE 7:**
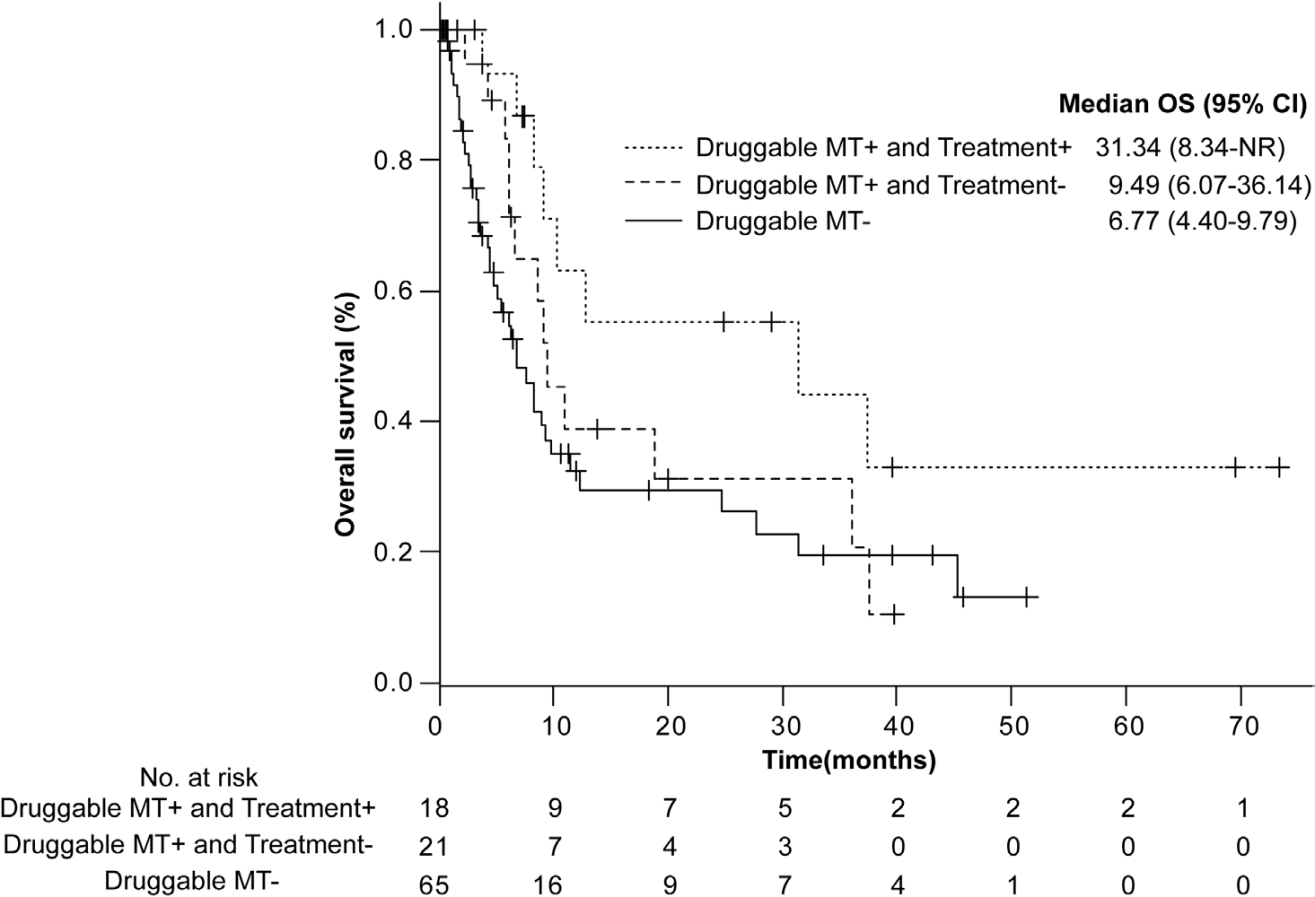
Post‐expert panel overall survival according to druggable genomic aberration status and receipt of recommended treatment. (1) Patients without druggable genomic aberrations (Druggable MT−), (2) patients with druggable genomic aberrations who did not receive matched therapy (Druggable MT+/Treatment−), and (3) patients with druggable genomic aberrations who received matched therapy (Druggable MT+/Treatment+).

Of the 108 patients enrolled in this study, actionable and druggable genomic aberrations were identified in 89 and 40 (37%) patients, respectively (Figure 6). Of the 38 patients (35.2%) for whom treatment was recommended by the EP, 14 (13%) were administered approved drugs, 19 were included in clinical trials, and five were administered off‐label drugs. Of these, only 18 (16.6%) patients received treatment (12 with approved drugs and six in clinical trials), and four were transferred to another hospital and could not be followed up. Only 11 patients (10.2%) were judged to have clinically responded to treatment by the attending physician or showed tumor shrinkage on imaging studies. Table 2 highlights the correlation between the mutations identified in the 18 patients and specific treatments in clinical trials or approved drugs. Of the 108 patients, four died before the EP review and were excluded from the post‐EP survival analysis. A total of 104 patients were therefore included in the landmark analysis. Kaplan–Meier analysis demonstrated a difference in the post‐EP OS among the three groups, showing a trend toward statistical significance (log‐rank *p* = 0.054) (Figure 7). The median OS (95% CI) was 6.77 (range: 4.40–9.79) months in patients without druggable genomic aberrations, 9.49 (range: 6.07–36.14) months in patients with druggable genomic aberrations who did not receive the recommended treatment, and 31.34 (range: 8.34–not reached) months in patients with druggable genomic aberrations who received the recommended treatment.

**TABLE 2:** Druggable genomic aberrations and the type of treatment actually received and clinical outcomes.

Age	Sex	Brinkman index	Pre‐treatment lines, *N*	Histology	Pre‐defined driver mutations	Biomarker tests	Newly detected genomic aberration	Received treatment after EP	Approved drugs	Clinical response
49	M	240	2	Adeno	None	Single	ERBB2 snv	Clinical trial	—	PR
50	M	200	3	Adeno	None	Single	KRAS G12C	Clinical trial	—	PR
77	F	1350	3	Adeno	None	Single	EGFR L858R	Approved drug	Osimertinib	PR
68	M	0	8	Squamous	None	Not tested	EML4‐ALK	Approved drug	Alectinib	PR
45	M	0	1	Adeno	None	Single	ERBB2 ex20ins	Clinical trial	—	NA
32	F	0	4	Adeno	None	Single	KIF5B‐RET	Clinical trial	—	UK
43	F	0	2	Adeno	None	Single	CD74‐ROS1	Approved drug	Crizotinib	PR
50	F	0	4	Adeno	None	Single	MET ex14skip	Approved drug	Capmatinib	NA
75	F	0	4	Adeno	None	Single	MET ex14skip	Approved drug	Capmatinib	PR
49	F	0	2	Adeno	None	Single	EGFR uncommon	Approved drug	Osimertinib	PR
70	M	1350	5	Adeno	None	Single	BRAF V600E	Approved drug	Dabrafenib + trametinib	NE
70	F	0	1	Adeno	EGFR del19	Single	EGFR Del19, T790M	Approved drug	Osimertinib	UK
63	F	0	5	Adeno	None	Single	EGFRex19del	Approved drug	Alectinib	PR
54	M	560	2	Adeno	ALK‐Fusion	ODxTT‐M	ALK G1202R	Approved drug	Lorlatinib	SD
93	F	0	0	Adeno	None	Not tested	EGFR L858R	Approved drug	Osimertinib	PR
60	F	0	3	Adeno	None	ODxTT‐M	ERBB2 ex20ins	Clinical trial	—	UK
66	M	570	2	Adeno	ALK‐Fusion	ODxTT‐M	ALK‐fusion+G1202R	Approved drug	Lorlatinib	PR
66	F	0	2	Adeno	None	Single	CD74‐NRG1 fusion	Not matched clinical Trial	—	NA

Abbreviations: ALK, anaplastic lymphoma kinase; EGFR, epidermal growth factor receptor; F, female; HER2, human epidermal growth factor receptor 2; M, male; MET, mesenchymal–epithelial transition factor; NA, not‐available; NE, not evaluable for response; ODxTT‐M, Oncomine Dx Target Test Multi‐CDx; PR, partial response; ROS1, c‐ros oncogene 1; SD, stable disease.

## 4 Discussion

In this study, we evaluated the usefulness of CGP testing in patients with NSCLC in clinical practice. Notably, druggable genetic mutations were found in 37% of the patients. Treatment options were proposed for 35.2% of the patients, and an approved drug was recommended in 13% of the cases, with most patients (85.7%) undergoing therapeutic intervention. However, participation in clinical trials was low (31.6%) and no patients had received off‐label treatments, thus identifying barriers to accessing these treatments.

The percentage of druggable gene mutations detected in CGP tests depends on the patient population and test method used; however, it is generally found in approximately 36%–62.9% of patients with solid tumors [9, 10]. This percentage varies widely among cancer types [10, 11]. In Japan, Ida et al. reported that 46.9% (196 of 418) of all cases tested with F1CDx or NCC OncoPanel had a druggable genomic alteration and further noted that the rate of common cancers was 59.5%, which was significantly higher than 38% in non‐common cancers [11]. There are few reports on the proportion of druggable genetic mutations in NSCLC; in our study, the proportion was 37%. The major difference between NSCLC and other cancer types is that the likelihood of finding a driver mutation for which an approved drug is indicated is relatively high [11]. Sunami et al. [12] reported that among 26 patients with NSCLC, 12 (46%) harbored FDA‐ or PMDA‐approved driver mutations for the tumor type. This high detection rate may be because biomarker testing has not been well validated in routine practice for NSCLC; this was before the approval of multiple biomarker testing in Japan. A recent study of Japanese nationwide CGP data found new driver gene mutations in 24.5% (81/330) of patients with NSCLC who initially tested negative for EGFR, ALK, ROS1, and BRAF [13]. Furthermore, the report is noteworthy in that EGFR mutations were the most frequently detected among these mutations. This trend was also observed in the current study, with newly detected EGFR mutations identified in 10.3% of patients who were initially tested negative by conventional biomarker testing. Thus, while CGP testing is important in terms of the opportunity to provide molecular targeted drugs to patients with NSCLC, the fact that these mutations are not detected by biomarker tests performed in routine clinical practice is also a concern. Importantly, our study provides real‐world data focusing specifically on NSCLC patients who underwent CGP testing after completion of standard treatment, which remains insufficiently characterized in previous reports.

In a previous study, we compared the performance of the ODxTT‐M and Cobas EGFR Mutation Test v2 (Cobas EGFR; Roche Molecular Systems) for the detection of EGFR mutations [14]. Our findings revealed a 2.5% discrepancy between the two tests, which was largely attributed to differences in the variants that could be identified. The ODxTT‐M, which simultaneously evaluates 46 cancer‐related genes, including nine therapeutic target genes, is the first NGS panel for NSCLC testing to be approved for insurance coverage in Japan (approval received in June 2019). ODxTT‐M is an NGS‐based test method that employs an amplicon sequence‐based hotspot panel test. This method amplifies each target site using primers that span part of the coding region. Consequently, as with PCR‐based testing methods, ODxTT‐M is only capable of detecting mutations in targeted mutation hotspots. The F1CDx and NCC OncoPanel have been approved as CGP tests that utilize the hybrid capture method. Each panel can detect mutations, amplifications, and homozygous deletions across all coding regions of the target gene, in addition to oncogene rearrangements. Consequently, F1CDx and NCC OncoPanel can detect rare mutations that are not discernible by ODxTT‐M or PCR‐based hotspot panel test employing hybrid capture methods. Moreover, this phenomenon is not exclusive to EGFR, and the current ODxTT‐M assay may not detect uncommon fusion partners or variants of ALK and ROS1. As previously reported, one patient included in this study exhibited an ROS1‐CD74 fusion variant (CD74 exon 3 fused to ROS1 exon 34) that was undetectable by conventional RT‐PCR‐based testing [15]. This tumor demonstrated responsiveness to the ROS1 inhibitor crizotinib, indicating oncogenic activity of this novel fusion variant.

The correlation between the frequency of driver gene mutations and patient background exhibited some variation by gene; however, it was notably higher for EGFR mutations, ALK fusions, ROS1 fusions, and HER2 mutations in women, non‐smokers, and adenocarcinomas. Furthermore, EGFR mutations are more frequently observed in Asian populations, with EGFR positivity reported in 50% of the NSCLC cases in Japan. The results from the nationwide CGP data also showed a 66% detection rate of driver gene mutations in non‐smokers, which was higher than the 36% detection rate in smokers [13]. However, this report was based on the overall population and not on newly detected driver gene mutations. The overall percentage of newly detected druggable mutations in this study was 37%, but it was significantly higher in non‐smokers (55.6%) compared with smokers (27.8%), although the percentage did not vary by sex, age, or histological type. Considering the epidemiological data and findings of our study, it can be posited that CGP testing for NSCLC would be more beneficial for non‐smokers.

There have been multiple reports of resistance mutations following the administration of molecular targeted drug therapies for NSCLC. In the case of EGFR, the frequency of the T790M mutation, which makes it resistant to first‐ and second‐generation EGFR‐TKIs, is approximately 50%. Osimertinib, a third‐generation EGFR‐TKI, has demonstrated efficacy against T790M [16]. Additionally, reports have documented the emergence of resistance mutations following the administration of osimertinib as a first‐ or second‐line therapeutic option. The most common tertiary EGFR mutation is EGFR C797S, which occurs in exon 20 and accounts for 10%–26% of cases exhibiting resistance to osimertinib as a second‐line therapy [17]. When osimertinib is administered as a first‐line therapy, the C797S mutation occurs at a frequency of 7%, representing the second most common mechanism of drug resistance after MET amplification [18]. This study included 14 patients who received EGFR‐TKI treatment, 12 of whom received osimertinib. Among these, the C797S mutation was identified in three patients (25%), one with a tertiary EGFR mutation and two with a secondary EGFR mutation, after first‐line osimertinib treatment. The EGFR C797S mutation is a mechanism of osimertinib resistance; however, interestingly, preclinical data and case reports indicate that tumors may transiently respond to first‐generation EGFR‐TKIs (gefitinib or erlotinib) in this clinical scenario [19]. CGP testing following osimertinib resistance would be clinically beneficial to elucidate the mechanisms of resistance to EGFR‐TKIs and potentially provide an approved drug such as a first‐generation EGFR‐TKI.

Resistance to ALK inhibitors is mostly due to secondary on‐target mutations in ALK, and ALK G1202R mutations have been reported most frequently following the use of second‐generation ALK inhibitors [20, 21]. Two patients demonstrated ALK resistance following alectinib or brigatinib administration, as indicated by the presence of ALK G1202R. In these instances, lorlatinib was proposed as a potential therapeutic option, given its demonstrated efficacy against the ALK G1202R mutation. Additionally, ALK and MET amplification are rare mechanisms of resistance to second‐generation ALK TKIs; however, the precise frequency remains uncertain. The ability to sequence ALK inhibitors after use without confirming resistance‐on‐target mutations and the lack of a probable therapeutic strategy for resistance due to the activation of bypass signaling pathways limit the significance of CGP testing for ALK‐resistant patients. However, CGP testing can still play a crucial role in identifying rare resistance mechanisms and in guiding the selection of subsequent therapies, particularly as new targeted treatments continue to be developed.

Two reports from the National Cancer Center in Japan, published at different times, indicated that 12.2% and 13.4% of patients received genetically matched treatment. This percentage was significantly higher in patients with common cancers (16.2%) than in those with less common cancers (9.4%). Of particular note was the treatment of lung cancer, which accounted for 26.3% (10/38) of patients [11, 12]. Two previous reports also demonstrated that approved drugs are used as matched therapies in numerous cases of NSCLC. The proportion of matched patients in this study was 16.6% (18 of 108 patients), 12 of whom received the approved drug. This trend is consistent with that observed in previous reports. Nine patients (75%) treated with the approved drugs experienced a clinically meaningful treatment effect, either partial response (PR) or stable disease (SD).

In addition to response‐based outcomes, we conducted a landmark analysis of OS using the EP review date as the reference time point. Patients who received recommended treatment showed numerically prolonged post‐EP survival compared with the other groups. Although the difference did not reach a conventional statistical significance (log‐rank *p* = 0.054), the separation of survival curves may suggest a clinically meaningful trend toward improved outcomes associated with CGP‐guided treatment. These findings should be interpreted cautiously due to the retrospective design, limited sample size, and potential selection bias inherent in the landmark approach.

Participation in clinical trials to receive treatment with a new investigational drug is another avenue for receiving matched treatment. In our study, 31.6% (6 of 19 patients) of patients participated in the clinical trial. Compared with the percentage of patients who received the approved drugs, the percentage of patients participating in clinical trials was low. Clinical trials have strict eligibility criteria for selecting participants; if a patient's condition, age, treatment history, or general condition do not meet the criteria, they cannot participate in the trial. This may particularly affect patients who have undergone multiple treatments prior to CGP testing or who have experienced a decline in their clinical condition. To address this issue, it is essential that CGP testing is included as early as possible in a patient's treatment plan. In our study, five patients were recommended off‐label drugs by the EP; however, none received treatment. The low rate of treatment with off‐label drugs may be due to a combination of regulatory, financial, clinical, and logistical factors. This trend is observed not only in Japan, but also in other countries. In many healthcare systems, including those in the U.S. and Japan, regulatory approval and insurance coverage are strictly linked to approved drug indications. Off‐label use may not be covered by insurance, which results in high out‐of‐pocket costs for patients. Off‐label treatments are based on promising, but limited, clinical evidence. Access to off‐label treatments remains a complex challenge in precision oncology, despite the potential benefits of advanced genomic testing.

Several barriers may limit the clinical implementation of genomically matched therapies. First, the eligibility criteria for clinical trials are often strict, and patients with poor performance status or extensive prior treatments may not meet these requirements. Second, deterioration in general condition during the interval between CGP testing and treatment decision may prevent trial enrollment. Third, regulatory and financial constraints may restrict access to off‐label treatments, particularly when insurance coverage is not available. Finally, the geographic availability of clinical trials and investigational drugs may also limit patient participation. These factors collectively contribute to the relatively low rate of matched therapies observed in real‐world clinical practice.

This study has several limitations. First, this was a retrospective single‐center study with a limited number of cases, which inherently restricts the generalizability of the findings. In addition, because CGP testing was performed at the discretion of the attending physician and depended on tissue availability, selection and channeling biases may have influenced patient selection. Consequently, not all patients may have been equally considered for CGP testing, and the extent to which genetic mutations might have been missed by existing companion diagnostic tests remains uncertain. Second, although we performed a landmark analysis of overall survival, this analysis has several inherent limitations. The retrospective design, small sample size, and potential selection bias may have influenced the survival outcomes. In addition, the landmark approach may have introduced bias by excluding patients who died before expert panel review. Therefore, the observed survival differences should be interpreted with caution. Nevertheless, CGP testing identified previously undetected genomic alterations in a subset of patients and provided potential opportunities for targeted treatment that might not otherwise have been considered.

In conclusion, our study suggests that CGP testing in NSCLC patients may be beneficial in two ways: it may identify genomic mutations that have not been detected by companion diagnostics, and it may confirm resistance after treatment with molecular targeted drugs for driver gene mutations. In addition, the diagnostic utility of CGP testing for identifying treatable genomic mutations was higher in non‐smokers and was unaffected by histology or age.

## Author Contributions

**Shuji Murakami:** conceptualization, data curation, formal analysis, writing – original draft, writing – review and editing. **Yukihiko Hiroshima:** methodology, writing – review and editing. **Haruhiro Saito:** data curation, writing – review and editing. **Terufumi Kato:** data curation, writing – review and editing. **Seigo Katakura:** data curation, writing – review and editing. **Tetsuro Kondo:** data curation, writing – review and editing. **Tomoyuki Yokose:** methodology, writing – review and editing.

## Funding

The authors have nothing to report.

## Ethics Statement

This study was approved by the Kanagawa Cancer Centre Institutional Review Board (No. 2024 Eki‐58). The requirement for informed consent was waived due to the retrospective observational nature of the study.

## Conflicts of Interest

**Shuji Murakami:** Grants or contracts to institution: AstraZeneca, MSD, Daiichi Sankyo, Ono Pharmaceutical, Janssen Pharma, Chugai, Taiho, BeiGene, Novartis, and Amgen. **Honoraria for speaker to self:** AstraZeneca, Chugai Pharma, Eli Lilly, Takeda, and Merck. **Yukihiko Hiroshima, Tetsuro Kondo, and Tomoyuki Yokose:** Nothing to disclose. **Seigo Katakura:** Grants or contracts to institution: MSD, Eli Lilly, and Amgen. **Terufumi Kato:** Grants or contracts to institution: AstraZeneca, MSD, Blueprint Medicines, Daiichi‐Sankyo, GlaxoSmithKline, Janssen Pharma, Chugai, Bayer, Bristol‐Meyers Squibb, Boehringer Ingelheim, Haihe, Regeneron, and Medpace Japan. **Honoraria for speaker to self:** AstraZeneca, Chugai, Daiichi‐Sankyo, and Merck KGaA. **Haruhiro Saito:** Grants or contracts to institution: Daiichi‐Sankyo, Taiho, Ono Pharmaceutical, Pfizer, and Meck. **Honoraria for speaker to self:** AstraZeneca.

## Data Availability

The data that support the findings of this study are available from the corresponding author upon reasonable request.

## References

[1] R. S. Herbst , D. Morgensztern , and C. Boshoff , “The Biology and Management of Non‐Small Cell Lung Cancer,” Nature 553 (2018): 446–454. 29364287 10.1038/nature25183

[2] M. G. Kris , B. E. Johnson , L. D. Berry , et al., “Using Multiplexed Assays of Oncogenic Drivers in Lung Cancers to Select Targeted Drugs,” Journal of the American Medical Association 311 (2014): 1998–2006. 24846037 10.1001/jama.2014.3741 PMC4163053

[3] T. Sakamoto , T. Matsubara , T. Takahama , et al., “Biomarker Testing in Patients With Unresectable Advanced or Recurrent Non‐Small Cell Lung Cancer,” JAMA Network Open 6 (2023): e2347700. 38100106 10.1001/jamanetworkopen.2023.47700 PMC10724778

[4] Y. Yatabe , K. Sunami , K. Goto , et al., “Multiplex Gene‐Panel Testing for Lung Cancer Patients,” Pathology International 70 (2020): 921–931. 32956529 10.1111/pin.13023

[5] S. Murakami , K. Shinada , Y. Otsutsumi , et al., “Comparison Between Next‐Generation Sequencing and Multiplex Polymerase Chain Reaction Assays for Nonsmall‐Cell Lung Cancer Molecular Diagnosis,” Cancer Medicine 13 (2024): e7162. 38572952 10.1002/cam4.7162 PMC10993699

[6] K. Kunimasa , S. Matsumoto , T. Kawamura , et al., “Clinical Application of the AMOY 9‐in‐1 Panel to Lung Cancer Patients,” Lung Cancer 179 (2023): 107190. 37058787 10.1016/j.lungcan.2023.107190

[7] Y. Naito , H. Aburatani , T. Amano , et al., “Clinical Practice Guidance for Next‐Generation Sequencing in Cancer Diagnosis and Treatment (Edition 2.1),” International Journal of Clinical Oncology 26 (2021): 233–283. 33249514 10.1007/s10147-020-01831-6 PMC7819967

[8] Y. Kanda , “Investigation of the Freely Available Easy‐to‐Use Software ‘EZR’ for Medical Statistics,” Bone Marrow Transplantation 48 (2013): 452–458. 23208313 10.1038/bmt.2012.244 PMC3590441

[9] A. Zehir , R. Benayed , R. H. Shah , et al., “Mutational Landscape of Metastatic Cancer Revealed From Prospective Clinical Sequencing of 10,000 Patients,” Nature Medicine 23 (2017): 703–713. 10.1038/nm.4333 PMC5461196 28481359

[10] T. Kondo , J. Matsubara , P. N. Quy , et al., “Comprehensive Genomic Profiling for Patients With Chemotherapy‐Naive Advanced Cancer,” Cancer Science 112 (2021): 296–304. 33007138 10.1111/cas.14674 PMC7780032

[11] H. Ida , T. Koyama , T. Mizuno , et al., “Clinical Utility of Comprehensive Genomic Profiling Tests for Advanced or Metastatic Solid Tumor in Clinical Practice,” Cancer Science 113 (2022): 4300–4310. 36106376 10.1111/cas.15586 PMC9746060

[12] K. Sunami , H. Ichikawa , T. Kubo , et al., “Feasibility and Utility of a Panel Testing for 114 Cancer‐Associated Genes in a Clinical Setting: A Hospital‐Based Study,” Cancer Science 110 (2019): 1480–1490. 30742731 10.1111/cas.13969 PMC6447843

[13] M. Ishida , M. Iwasaku , T. Doi , et al., “Nationwide Data From Comprehensive Genomic Profiling Assays for Detecting Driver Oncogenes in Non‐Small Cell Lung Cancer,” Cancer Science 115 (2024): 1656–1664. 38450844 10.1111/cas.16130 PMC11093184

[14] S. Murakami , T. Yokose , K. Shinada , et al., “Comparison of Next‐Generation Sequencing and Cobas EGFR Mutation Test v2 in Detecting EGFR Mutations,” Thoracic Cancer 13 (2022): 3217–3224. 36203199 10.1111/1759-7714.14685 PMC9663664

[15] M. H. Hashiguchi , T. Sato , R. Watanabe , et al., “A Case of Lung Adenocarcinoma With a Novel CD74‐ROS1 Fusion Variant Identified by Comprehensive Genomic Profiling That Responded to Crizotinib and Entrectinib,” Thoracic Cancer 12 (2021): 2504–2507. 34319660 10.1111/1759-7714.14093 PMC8447907

[16] G. Goss , C. M. Tsai , F. A. Shepherd , et al., “Osimertinib for Pretreated EGFR Thr790Met‐Positive Advanced Non‐Small‐Cell Lung Cancer (AURA2): A Multicentre, Open‐Label, Single‐Arm, Phase 2 Study,” Lancet Oncology 17 (2016): 1643–1652. 27751847 10.1016/S1470-2045(16)30508-3

[17] T. S. Mok , Y. L. Wu , M. J. Ahn , et al., “Osimertinib or Platinum‐Pemetrexed in EGFR T790M‐Positive Lung Cancer,” New England Journal of Medicine 376 (2017): 629–640. 27959700 10.1056/NEJMoa1612674 PMC6762027

[18] A. Leonetti , S. Sharma , R. Minari , P. Perego , E. Giovannetti , and M. Tiseo , “Resistance Mechanisms to Osimertinib in EGFR‐Mutated Non‐Small Cell Lung Cancer,” British Journal of Cancer 121 (2019): 725–737. 31564718 10.1038/s41416-019-0573-8 PMC6889286

[19] D. Rangachari , C. To , J. E. Shpilsky , et al., “EGFR‐Mutated Lung Cancers Resistant to Osimertinib Through EGFR C797S Respond to First‐Generation Reversible EGFR Inhibitors but Eventually Acquire EGFR T790M/C797S in Preclinical Models and Clinical Samples,” Journal of Thoracic Oncology 14 (2019): 1995–2002. 31377341 10.1016/j.jtho.2019.07.016 PMC6823139

[20] J. F. Gainor , L. Dardaei , S. Yoda , et al., “Molecular Mechanisms of Resistance to First‐ and Second‐Generation ALK Inhibitors in ALK‐Rearranged Lung Cancer,” Cancer Discovery 6 (2016): 1118–1133. 27432227 10.1158/2159-8290.CD-16-0596 PMC5050111

[21] N. Yanagitani , K. Uchibori , S. Koike , et al., “Drug Resistance Mechanisms in Japanese Anaplastic Lymphoma Kinase‐Positive Non‐Small Cell Lung Cancer and the Clinical Responses Based on the Resistant Mechanisms,” Cancer Science 111 (2020): 932–939. 31961053 10.1111/cas.14314 PMC7060465

